# Supplementary material for: Engagement and retention in HIV care in rural Southern U.S. using an All-Payer Claims Database
Source: PLoS One. 2025 Dec 30;20(12):e0339520. doi: 10.1371/journal.pone.0339520 (PMC12753065; doi:10.1371/journal.pone.0339520)
Supplement: S1 Table — (DOCX) [file pone.0339520.s001.docx]

**S1 Table.** **HIV testing and engagement in the HIV care continuum in Arkansas, 2013-2017.**

|  | **HIV test** | | **People living with HIV/AIDS (PLWHA)** | | | | | | | | | | | | | | | |
| --- | --- | --- | --- | --- | --- | --- | --- | --- | --- | --- | --- | --- | --- | --- | --- | --- | --- | --- |
|  | **N**  **(test)** | **N**  **(person)** | **N** | **N*** | **Engaged in HIV care** | | **Retained in HIV care** | | **Picked up prescription(s)** | | | **Had CD4 test(s)** | | | **Had viral load test(s)** | | **Had general clinical visit(s)** |  |
|  |  |  |  |  | **N (%)** | **p** | **N (%)** | **p** | **N (%)** | **p** | **N (%)** | | **p** | **N (%)** | | **p** | **N (%)** | **p** |
| **Gender** |  |  |  |  |  |  |  |  |  |  |  | |  |  | |  |  |  |
| Male | 53329 | 32220 | 4078 | 3443 | 2036 (59.1%) | <.001 | 1272 (36.94%) | <.001 | 2940 (85.4%) | <.001 | 903 (26.2%) | | <.001 | 2005 (58.2%) | | <.001 | 3134 (91%) | <.001 |
| Female | 181561 | 121336 | 1821 | 1590 | 741 (46.6%) |  | 482 (30.31%) |  | 1123 (70.6%) |  | 333 (20.9%) | |  | 723 (45.5%) | |  | 1438 (90.4%) |  |
| **Age groups** |  |  |  |  |  |  |  |  |  |  |  | |  |  | |  |  |  |
| <13 | 3352 | 2328 | 153 | 66 | 18 (27.3%) | <.001 | 9 (13.64%) | <.001 | 45 (67.9%) | <.001 | 2 (3%) | | <.001 | 17 (25.8%) | | <.001 | 35 (53%) | <.001 |
| 13-14 | 2666 | 1834 | 31 | 30 | 5 (16.7%) |  | 3 (10%) |  | 5 (17.2%) |  | 0 (0%) | |  | 5 (16.7%) | |  | 28 (93.3%) |  |
| 15-24 | 83616 | 54791 | 435 | 386 | 230 (59.6%) |  | 133 (34.46%) |  | 339 (87.8%) |  | 94 (24.4%) | |  | 235 (60.9%) | |  | 379 (98.2%) |  |
| 25-34 | 75621 | 50673 | 1117 | 937 | 620 (66.2%) |  | 363 (38.74%) |  | 898 (95.8%) |  | 278 (29.7%) | |  | 609 (65%) | |  | 911 (97.2%) |  |
| 35-44 | 30775 | 20110 | 1213 | 1018 | 638 (62.7%) |  | 380 (37.33%) |  | 865 (85%) |  | 244 (24%) | |  | 612 (60.1%) | |  | 957 (94%) |  |
| 45-54 | 20675 | 12241 | 1598 | 1372 | 802 (58.5%) |  | 537 (39.14%) |  | 1105 (80.5%) |  | 375 (27.3%) | |  | 767 (55.9%) | |  | 1247 (90.9%) |  |
| 55-64 | 14959 | 9422 | 998 | 893 | 378 (42.3%) |  | 276 (30.91%) |  | 617 (69.1%) |  | 194 (21.7%) | |  | 392 (43.9%) | |  | 706 (79.1%) |  |
| >64 | 3222 | 2154 | 354 | 331 | 86 (26%) |  | 54 (16.31%) |  | 189 (57.1%) |  | 49 (14.8%) | |  | 91 (27.5%) | |  | 309 (93.4%) |  |
| **Year**** |  |  |  |  |  |  |  |  |  |  |  | |  |  | |  |  |  |
| 2013 | 62525 | 51040 | 1803 | 1533 | 739 (48.2%) | .035 (up) | 674 (43.97%) | .17 | 937 (61.1%) | <.001 (up) | 302 (19.7%) | | .05 (down) | 726 (47.4%) | | .004 (up) | 1036 (67.6%) | <.001 (up) |
| 2014 | 53058 | 45169 | 2639 | 2048 | 1021 (49.9%) |  | 961 (46.92%) |  | 1604 (78.3%) |  | 436 (21.3%) | |  | 999 (48.8%) | |  | 1403 (68.5%) |  |
| 2015 | 45729 | 37747 | 3028 | 2514 | 1300 (51.7%) |  | 1150 (45.74%) |  | 1971 (78.4%) |  | 530 (21.1%) | |  | 1290 (51.3%) | |  | 1778 (70.7%) |  |
| 2016 | 39115 | 30541 | 3485 | 2924 | 1535 (52.5%) |  | 1333 (45.59%) |  | 2470 (84.5%) |  | 612 (20.9%) | |  | 1497 (51.2%) | |  | 2130 (72.8%) |  |
| 2017 | 34465 | 25173 | 3761 | 3186 | 1662 (52.2%) |  | 1396 (43.82%) |  | 2811 (88.2%) |  | 585 (18.4%) | |  | 1623 (50.9%) | |  | 2318 (72.8%) |  |
| **Rural /urban** |  |  |  |  |  |  |  |  |  |  |  | |  |  | |  |  |  |
| mostly urban | 154130 | 99769 | 4322 | 3710 | 2074 (55.9%) | .19 | 1297 (34.96%) | .58 | 2960 (79.8%) | .007 | 927 (25%) | | .04 | 2033 (54.8%) | | .36 | 3343 (90.1%) | <.001 |
| mostly rural | 69849 | 46762 | 1419 | 1183 | 631 (53.3%) |  | 415 (35.08%) |  | 992 (83.9%) |  | 287 (24.3%) | |  | 623 (52.7%) | |  | 1107 (93.6%) |  |
| completely rural | 10259 | 7027 | 158 | 140 | 72 (51.4%) |  | 43 (30.71%) |  | 111 (79.3%) |  | 22 (15.7%) | |  | 72 (51.4%) | |  | 122 (87.1%) |  |
| **Insurance** |  |  |  |  |  |  |  |  |  |  |  | |  |  | |  |  |  |
| Medicaid | 114372 | 69528 | 2831 | 2063 | 1017 (49.3%) | <.001 | 668 (32.38%) | <.001 | 1486 (72%) | <.001 | 461 (22.3%) | | <.001 | 991 (48%) | | <.001 | 1999 (96.9%) | <.001 |
| Medicare | 3347 | 2226 | 733 | 667 | 171 (25.6%) |  | 98 (14.69%) |  | 627 (94%) |  | 84 (12.6%) | |  | 169 (25.3%) | |  | 273 (40.9%) |  |
| private | 116890 | 81803 | 2335 | 2303 | 1589 (69%) |  | 989 (42.94%) |  | 1947 (84.5%) |  | 691 (30%) | |  | 1568 (68.1%) | |  | 2300 (99.9%) |  |

*Number of PLWHA who had one full calendar year of insurance coverage

**Cochran-Armitage Trend Test was used for trend analysis over the years; chi-sq test was used for analyzing all other variables
