# Supplementary material for: Engagement and retention in HIV care in rural Southern U.S. using an All-Payer Claims Database
Source: PLoS One. 2025 Dec 30;20(12):e0339520. doi: 10.1371/journal.pone.0339520 (PMC12753065; doi:10.1371/journal.pone.0339520)
Supplement: Attachment — Submitted filename: Response to Reviewers.docx [file pone.0339520.s002.docx]

**Response to Reviewers**

When submitting your revision, we need you to address these additional requirements.

1. Please ensure that your manuscript meets PLOS ONE's style requirements, including those for file naming. The PLOS ONE style templates can be found at

https://journals.plos.org/plosone/s/file?id=wjVg/PLOSOne_formatting_sample_main_body.pdf and

<https://journals.plos.org/plosone/s/file?id=ba62/PLOSOne_formatting_sample_title_authors_affiliations.pdf>

**Response**: Thank you for these helpful resources. We have updated the manuscript to be in alignment with PLOS ONE’s style requirements.

2. Thank you for stating the following financial disclosure:

[The study was supported by National Institute of Health (NIH) grants K01MH19660, R25MH083620, and the Providence/Boston Center for AIDS Research (P30AI042853).].

Please state what role the funders took in the study. If the funders had no role, please state: "The funders had no role in study design, data collection and analysis, decision to publish, or preparation of the manuscript."

If this statement is not correct you must amend it as needed.

Please include this amended Role of Funder statement in your cover letter; we will change the online submission form on your behalf.

**Response**: Thank you for your input. We have added this sentence to the cover letter.

3. For studies involving third-party data, we encourage authors to share any data specific to their analyses that they can legally distribute. PLOS recognizes, however, that authors may be using third-party data they do not have the rights to share. When third-party data cannot be publicly shared, authors must provide all information necessary for interested researchers to apply to gain access to the data. (https://journals.plos.org/plosone/s/data-availability#loc-acceptable-data-access-restrictions)

For any third-party data that the authors cannot legally distribute, they should include the following information in their Data Availability Statement upon submission:

1) A description of the data set and the third-party source

Thank you - we have updated the Data availability statement to include a description of the source, which is the Arkansas All-Payer Claims Database, managed by the Arkansas Center for Health Improvement.

2) If applicable, verification of permission to use the data set

3) Confirmation of whether the authors received any special privileges in accessing the data that other researchers would not have

4) All necessary contact information others would need to apply to gain access to the data

**Response**: Thank you - we have updated the Data availability statement to include a description of the source, which is the Arkansas All-Payer Claims Database, managed by the Arkansas Center for Health Improvement, including contact information. This data is not available for sharing, it must be purchased or obtained directly through the AR APCD. The authors did not receive any special privileges in accessing the data that other researchers would not have.

4. We note that Figures 3 and 4 in your submission contain [map/satellite] images which may be copyrighted. All PLOS content is published under the Creative Commons Attribution License (CC BY 4.0), which means that the manuscript, images, and Supporting Information files will be freely available online, and any third party is permitted to access, download, copy, distribute, and use these materials in any way, even commercially, with proper attribution. For these reasons, we cannot publish previously copyrighted maps or satellite images created using proprietary data, such as Google software (Google Maps, Street View, and Earth). For more information, see our copyright guidelines: http://journals.plos.org/plosone/s/licenses-and-copyright.

We require you to either (1) present written permission from the copyright holder to publish these figures specifically under the CC BY 4.0 license, or (2) remove the figures from your submission:

1. You may seek permission from the original copyright holder of Figures 3 and 4 to publish the content specifically under the CC BY 4.0 license.

We recommend that you contact the original copyright holder with the Content Permission Form (http://journals.plos.org/plosone/s/file?id=7c09/content-permission-form.pdf) and the following text:

“I request permission for the open-access journal PLOS ONE to publish XXX under the Creative Commons Attribution License (CCAL) CC BY 4.0 (http://creativecommons.org/licenses/by/4.0/). Please be aware that this license allows unrestricted use and distribution, even commercially, by third parties. Please reply and provide explicit written permission to publish XXX under a CC BY license and complete the attached form.”

Please upload the completed Content Permission Form or other proof of granted permissions as an "Other" file with your submission.

In the figure caption of the copyrighted figure, please include the following text: “Reprinted from [ref] under a CC BY license, with permission from [name of publisher], original copyright [original copyright year].”

2. If you are unable to obtain permission from the original copyright holder to publish these figures under the CC BY 4.0 license or if the copyright holder’s requirements are incompatible with the CC BY 4.0 license, please either i) remove the figure or ii) supply a replacement figure that complies with the CC BY 4.0 license. Please check copyright information on all replacement figures and update the figure caption with source information. If applicable, please specify in the figure caption text when a figure is similar but not identical to the original image and is therefore for illustrative purposes only.

The following resources for replacing copyrighted map figures may be helpful:

USGS National Map Viewer (public domain): http://viewer.nationalmap.gov/viewer/

The Gateway to Astronaut Photography of Earth (public domain): http://eol.jsc.nasa.gov/sseop/clickmap/

Maps at the CIA (public domain): https://www.cia.gov/library/publications/the-world-factbook/index.html and https://www.cia.gov/library/publications/cia-maps-publications/index.html

NASA Earth Observatory (public domain): http://earthobservatory.nasa.gov/

Landsat: http://landsat.visibleearth.nasa.gov/

USGS EROS (Earth Resources Observatory and Science (EROS) Center) (public domain): http://eros.usgs.gov/#

Natural Earth (public domain): <http://www.naturalearthdata.com/>

**Response**: These images were made with ArcGIS pro, which MG has access to use through University of Arkansas for Medical Sciences. No copyright permission is needed.

5. If the reviewer comments include a recommendation to cite specific previously published works, please review and evaluate these publications to determine whether they are relevant and should be cited. There is no requirement to cite these works unless the editor has indicated otherwise.

**Response**: Thank you for this comment.

6. Please review your reference list to ensure that it is complete and correct. If you have cited papers that have been retracted, please include the rationale for doing so in the manuscript text, or remove these references and replace them with relevant current references. Any changes to the reference list should be mentioned in the rebuttal letter that accompanies your revised manuscript. If you need to cite a retracted article, indicate the article’s retracted status in the References list and also include a citation and full reference for the retraction notice.

**Response**: Thank you for this comment. All references have been checked.

Additional Editor Comments:

Thank you for your scholarly contribution. I'd also like to thank the authors for choosing PLOS ONE to publish your findings from this study. Comments from reviewers are provided below. Please review these comments and I suggest address them and resubmit your manuscript. Your timely response would help this study be published and will make it accessible to interested readers across the world. I look forward to reviewing your revised manuscript. I wish you good luck with your future endeavors.


[Note: HTML markup is below. Please do not edit.]

Reviewers' comments:

Reviewer's Responses to Questions

**Comments to the Author**

1. Is the manuscript technically sound, and do the data support the conclusions?

The manuscript must describe a technically sound piece of scientific research with data that supports the conclusions. Experiments must have been conducted rigorously, with appropriate controls, replication, and sample sizes. The conclusions must be drawn appropriately based on the data presented.

Reviewer #1: Yes

Reviewer #2: Yes

2. Has the statistical analysis been performed appropriately and rigorously?

Reviewer #1: Yes

Reviewer #2: I Don't Know

3. Have the authors made all data underlying the findings in their manuscript fully available?

The [PLOS Data policy](http://www.plosone.org/static/policies.action#sharing) requires authors to make all data underlying the findings described in their manuscript fully available without restriction, with rare exception (please refer to the Data Availability Statement in the manuscript PDF file). The data should be provided as part of the manuscript or its supporting information, or deposited to a public repository. For example, in addition to summary statistics, the data points behind means, medians and variance measures should be available. If there are restrictions on publicly sharing data—e.g. participant privacy or use of data from a third party—those must be specified.

Reviewer #1: Yes

Reviewer #2: Yes

4. Is the manuscript presented in an intelligible fashion and written in standard English?

PLOS ONE does not copyedit accepted manuscripts, so the language in submitted articles must be clear, correct, and unambiguous. Any typographical or grammatical errors should be corrected at revision, so please note any specific errors here.

Reviewer #1: Yes

Reviewer #2: Yes

5. Review Comments to the Author

Please use the space provided to explain your answers to the questions above. You may also include additional comments for the author, including concerns about dual publication, research ethics, or publication ethics. (Please upload your review as an attachment if it exceeds 20,000 characters)

Reviewer #1: Overall, this manuscript provided important insights into engagement and retention in HIV care in Arkansas using All Payer Claims Databases, which represents an alternative approach to assessing engagement and retention in the developed world. The limitations are appropriately acknowledged, and the results are presented cautiously without being overstated. The manuscript is also well structured and aligns well with research and publication ethics.
That said, I have a few observations that may help strengthen the study.
Focus of findings: While the study set out to assess engagement and retention in care, a significant portion of the findings and discussion focusses on HIV testing. Since HIV testing would typically precede engagement and retention in care, further clarification on how these results contribute to the primary outcomes of engagement and retention would be helpful.

**Response:** Thank you for this comment. In the manuscript, we highlight how HIV testing is crucial for early diagnosis and linkage to care. Diagnosis is the first stage in the HIV care continuum.

Results section: under statistical methods, the manuscript mentions chi-square tests were used to examine meaningful differences; however, the supplementary table doesn't present the Chi-square results. Could the authors clarify whether these results are missing, presented elsewhere or were not statistically significant?
in summary, this is a well prepared manuscript that addresses an important topic in HIV care. These above few observations will improve the clarity and strength of the results.

**Response**: Thank you for this comment. Supplement Table 1, as well as the Results section, have been revised to show results of chi-square tests and Cochran-Armitage trend test for HIV care utilization.

Reviewer #2: This is a well written and valuable manuscript analyzing a data from the Arkansas All-payer Claims Database to characterize the HIV care cascade. Authors carefully document assumptions and criteria for extracting and coding cases. The study is important and worthy of publication. Authors are to be commended for developing a novel approach for exploring the HIV care continuum in Arkansas.

There are a number of minor issues that need to be addressed:

Throughout

PLoS One is a global publication, and ‘rural South’ needs to be explicitly unpacked both in the title and clearly in the introduction to the U.S. rural South.

**Response**: Thank you for this comment. We have updated the title and introduction to include rural Southern U.S.

Introduction

Please include supporting information such as the proportion of U.S. population in ‘the South’ and how you’re defining ‘the South’. Statistics about what proportion of the U.S. population lives in the south are needed to explain why it's problematic that >50% of PLWH in the U.S. live in the south.

**Response:** Thank you for this comment. We have updated the introduction to include the suggested information.

For a global readership, when statements such as “high rates of poverty (15.4% in 2017)” line 50 are made, it would be helpful to give the U.S. median or comparison given that many countries have far higher rates of poverty than 15.4%.

**Response:** Thank you for this comment. We have updated the introduction to include this statistic.

Line 65: ‘rapidly progressed’ - please define and provide a U.S. comparison for states where new HIV dx are associated with less rapid progression.

**Response:** Thank you for this comment. We have updated the introduction to include the suggested information.

Line 68 – ‘they must actively participate in care’ – there are PLWH who are virally suppressed and out of care; critical determinant is generally taking ART meds, not the entire chain of engagement you describe. Recommend rewording sentence to be more accurate.

**Response:** Thank you for this comment. We have reworded this sentence as suggested.

Please provide a sentence introducing the Ryan White program for readers who may not be familiar with it.

**Response:** Thank you for this comment. We have updated the introduction to include a definition of RWHAP.

Methods

Please define how you are using rural and urban since the definition varies across countries

**Response:** Thank you for this comment. We have included the rural and urban definitions in the Methods section.

Results

Line 192 – ‘ever tested for HIV’ – does this really mean ever tested, or does it mean tested during the four years captured by your data? Please be clear.

**Response:** Thank you for this comment. We have clarified that we meant testing during the four years captured by data.

Line 197 – ‘in mostly urban’ – does this mean areas where people live are predominantly urban? Or that most people are living in urban areas. The rural/urban transition spaces of suburbs are different, and it’s not clear from your language what you mean.

**Response:** Thank you for this comment. We have revised this sentence to be more clear.

Line 267 – ‘our analysis only reflects testing over the four year period covered in our dataset’

Limitations

You noted in lines 76-77 that approximately a third of PLWH in Arkansas receive care through the Ryan White program. A third is not ‘a small proportion’ (line 311). I recommend explicitly noting the approximately one third you’re potentially missing in the limitations (line 311), and also in the discussion (line 277-278) where you discuss the Ryan White program.

**Response:** Thank you for this comment. We have made the suggested revisions.

6. PLOS authors have the option to publish the peer review history of their article ([what does this mean?](https://journals.plos.org/plosone/s/editorial-and-peer-review-process#loc-peer-review-history)). If published, this will include your full peer review and any attached files.

If you choose “no”, your identity will remain anonymous but your review may still be made public.

**Do you want your identity to be public for this peer review?** For information about this choice, including consent withdrawal, please see our [Privacy Policy](https://www.plos.org/privacy-policy).

Reviewer #1: No

Reviewer #2: **Yes:** Debbie Humphries
